# Catastrophic pharmaceutical expenditure in patients with type 2 diabetes in Iran

**DOI:** 10.1186/s12939-022-01791-5

**Published:** 2022-12-29

**Authors:** Leila Zarei, Najmeh Moradi, Farzad Peiravian, Nazafarin Hatami-Mazinani, Motahareh Mahi-Birjand, Jalal Arabloo, Zaheer-Ud-Din Babar

**Affiliations:** 1 Assistant Professor in Pharmacoeconomics and Pharma Management, Health Policy Research Center, Institute of Health, Shiraz University of Medical Sciences, Shiraz, Fars Iran; 2 Assistant Professor in Pharmacoeconomics and Pharmaceutical Management, Health Management and Economics Research Center, Health Management Research Institute, Iran University of Medical Sciences, Tehran, Iran; 3 Assistant Professor in Pharmacoeconomics and Pharma Management, School of Pharmacy, Shahid Beheshti University of Medical Sciences, Tehran, Iran; 4 Department of Clinical Pharmacy, School of Pharmacy, Shiraz University of Medical Sciences, Shiraz, Iran; 5 Department of Clinical Pharmacy, School of Pharmacy, Infectious Diseases Research Center, Birjand University of Medical Sciences, Birjand, Iran; 6 Assistant Professor in Health Policy Making, Health Management and Economics Research Center, Iran University of Medical Sciences, Tehran, Iran; 7 Professor in Medicines and Healthcare. Department of Pharmacy, University of Huddersfield, Queensgate, HD1 3DH Huddersfield UK

**Keywords:** Diabetes, Catastrophic expenditures, Medication, Out of pocket, Middle-income countries

## Abstract

**Objectives:**

This study aimed to assess the financial burden of out-of-pocket (OOP) payments to purchase antidiabetic medicines for type 2 patients in Iran.

**Method:**

The “budget share” and “capacity to pay” approaches were employed to assess the catastrophic pharmaceutical expenditures of antidiabetic medication therapies. The catastrophic thresholds were adjusted for pharmaceutical sectors. The data was 2019 monthly household expenditures in rural and urban areas, insurance coverages of antidiabetic medicines and patients' out-of-pocket (OOP) payments in 30-day treatment schedules.

**Results:**

The results show that expenditure on diabetes medication therapies in the form of mono-dual therapy and some cases triple oral therapies were not catastrophic even for rural households. Insulin puts patients at risk of catastrophic pharmaceutical expenditures when added to the treatment schedules, and lack of financial protection intensifies it. In general, the poorer households and those resistant to first-line treatments were at increased risk of catastrophic pharmaceutical expenditures. The number of treatments that put patients at risk of catastrophic pharmaceutical expenditure in "budget share" was higher than the "capacity to pay" approach.

**Conclusions:**

Assessing medication treatment affordability instead of a single medicine assessment is needed. Assessment could be done by utilizing a macro-level data approach and applying adjusted pharmaceutical sector threshold values. Considering the variation between treatment schedules that put patients at risk of catastrophic pharmaceutical expenditures, targeted pharmaceutical policies and reimbursement decisions are recommended to promote Universal Health Coverage (UHC) and to protect vulnerable populations from hardship.

## Key points

- Defining specific adjusted catastrophic expenditure for the pharmaceutical sector based on national health systems whining countries is so critical.
- Using Macro-level data instead of conducting a household survey is recommended due to less expensive than the survey and feasibility of assessment in different time periods and facilitating international comparison between countries.
- Targeted pharmaceutical policies and reimbursement decisions are recommended to promote Universal Health Coverage (UHC) and protect vulnerable populations from hardship due to catastrophic pharmaceutical expenditures.

## Introduction

Many countries depend on the patients' out-of-pocket (OOP) payments in a healthcare financing system [1]. Excessive dependence on OOP due to disturbing the balance between patient income and expenses could lead to catastrophic expenditures and even impoverishment [2]. According to global estimates, 150 million individuals have catastrophic health expenditures (CHEs) each year, and approximately 100 million are moving toward poverty due to the OOP payments [3]. Both rich and developing nations experience CHEs; however, the most affected are those living in low-and middle-income countries (LMICs) [2].

Previous studies have shown that different components of health expenditures could create a financial burden for households and increase catastrophic expenditures, including household OOP payments on inpatient services, outpatient services, and medicines [4, 5]. Evidence shows that pharmaceutical expenditure typically accounts for the more significant share of OOP payments, ranging from 18–55% in developing countries [4]. In this way, the patients suffering from chronic non-communicable diseases (NCDs), including diabetes, where treatments extended to the patient's life span, face a higher risk of high prescription drug costs [6]. So, these individuals cannot purchase needed medicines and, in many instances, forgot about treatments or have to borrow to pay for the expenses [7].

Concerns regarding the lack of protection against catastrophic drug costs led political authorities and policymakers to assess the affordability of healthcare, improve access, and achieve Universal Health Coverage (UHC) [8]. Investigating the extent of catastrophic expenditure is the first step to developing an appropriate policy to ensure financial risk protection as the core of UHC.

Based on a systematic review, the incidence of CHEs in Iran was 7.5% (95% CI, 6.2–9.1] [9] between 1984 and 2014, 8.3% of CHEs were attributed to pharmaceutical expenditure [9]. In 2014, Iran launched the Health Transformation Plan (HTP) with the goals: of achieving UHC, improving access to health care, and reducing the households' OOP payments to < 30% and CHEs < 1%, [10]. However, only 12% of OOP payments for healthcare declined (from the previous 52% to the current 40%) [10]. Moreover, a study of per capita OOP revealed the most significant proportion of OOP payments spent on medicines before and after HTP [11]. This shows that pharmaceutical expenditures are among the leading causes of catastrophic expenditures [12]. Hence this study aims to fill this gap and provide an estimate of catastrophic pharmaceutical expenditures in Iran while investigating the expenditure of medication therapy in diabetes treatment in a comprehensive and novel approach.

Generally, there are three major concerns regarding earlier assessments and studies. First, previous studies in Iran [13–17] investigated the catastrophic effects of the limited number of essential medicines, including Metformin, glibenclamide, or Insulin, in monotherapy regimens [13, 14, 17]. However, in the real word, particularly in chronic diseases including diabetes, combination therapy is more common, i.e., dual or triple therapy [15, 16]. The current study assesses all possible antidiabetic medication therapies clinicians could prescribe.

Second, most studies conducted household surveys to collect data (micro-level) to measure CEs, which is difficult and costly. Previous studies have examined only one or a limited number of medicines [13–15]. The current study used aggregated data (macro-level) to measure CEs [8]. Third, in the majority of published studies, the general thresholds, which have been proposed to assess the CHEs, were cited. At the same time, as the PEs are one part of the total health expenditure (THEs), a specific adjusted threshold is needed. In the present study, an adjusted threshold has been used based on Iran's health system data.

## Materials and methods

### Study design

The present cross-sectional study assessed Iran's financial burden of OOP payments for diabetes medicines in 2019. This was done by utilizing well-accepted and commonly applied approaches and adjusting the thresholds for the pharmaceutical sector [18].

#### Catastrophic effect assessment of health expenditure

Catastrophic Health Expenditures (CHEs) refers to any medical treatment expenditure that can threaten a household's financial ability to maintain its subsistence needs [4]. In the health policy context, it is an indicator of financial protection [19]. Different methods are used to quantify the CHEs. The difference between the available methods is the definition of the household's resource capacity to pay for health care and the threshold value [18]. For example, in the "budget share approach" that was recommended by the World Bank (WB), Sustainable Development Goals (SDGs) and World Health Organization (WHO) [18], the household total expenditures were considered a denominator. If health expenditure exceeds the 10% and 25% threshold, these are regarded as CHEs [18, 20]. In the "capacity to pay" method,which was recommended by the WB, the total household expenditure minus actual food spending (it is called "actual food spending") was regarded as a denominator and if the health expenditures exceeded the threshold of 25% and 40% was considered as CHE [18].

#### Catastrophic pharmaceutical expenditures

Different components of health expenditure could create a financial burden for households and drive catastrophic expenditures. Most literature investigated catastrophic health expenditures as total -how much you spend on health during the last month- [4], and limited studies investigated catastrophic expenditures with a focus on its components [13, 15, 17, 21]. This is also the case for pharmaceutical expenditures; few studies have investigated it. Moreover, there is no recommended threshold in the literature for CPE. Therefore, to estimate catastrophic pharmaceutical expenditures as a part of total health expenditure in any health system, there is a need to adjust these thresholds.

By considering that pharmaceutical expenditure constitutes 20% of total health expenditure (THE) in Iran, we set new thresholds by multiplying this percentage by the predominantly used thresholds in literature for estimating Catastrophic Health expenditure (CHE) (10% or 40%). See Table 1:

**Table 1:** Adjusted threshold value for assessing catastrophic pharmaceutical expenditures in Iran

Approach	Standard Thresholds for estimating CHEs	Share of pharmaceutical expenditures from HE in Iran	Adjusted Thresholds for estimating CPEs*
Budget share	10%	20%	(0.20/0.10) = 2%
Capacity to pay	40%		(0.20/0.40) = 8%

^*^ *CPEs* Catastrophic pharmaceutical expenditures

#### The treatment schedule/treatment regimens

Most international clinical guidelines recommend monotherapy as the first-line treatment, including Metformin, Glibenclamide and Gliclazide. In the absence of optimal (appropriate) blood glucose levels, dual therapy was prescribed, i.e., Metformin plus one oral medication including Acarbose, Repaglinide, Glibenclamide or Pioglitazone. Triple therapy initiatives were prescribed when mono and dual-therapy failed, including Metformin plus two oral medications; Metformin plus basal Insulins or Insulin therapy.

### Data collection and analysis

This study employs the macro method suggested by Niëns et al. [8]. This method uses aggregated expenditure data instead of conducting a household survey to measure the catastrophic effects of medicines in diabetes treatment. In this approach, there is no sampling and survey at the household level. This method is less expensive than the survey, could be used in different periods, and facilitate international comparison between countries. This is due to access to macro data. In the current study, the hypothetical sample was a household in which one of the family members had type 2 diabetes in an urban or rural area with different income groups. The clinicians prescribed individualized treatment schedules regarding the patient's medical conditions.

The needed data on monthly household expenditure in rural and urban areas was obtained from the Statistical Center of Iran (SCI) [22]. The SCI is the national organization in Iran. Designing, planning and coordinating data production processes in various areas such as population, workforce, household income and expenditure are the major activities of the SCI.

To estimate monthly patients' out-of-pocket (OOP) payments, first, the medication therapy costs in each treatment scenario were estimated regarding treatment schedules in Table 2. Then the patient's medication therapy cost for the 30-day treatment course was calculated using the lowest generic price of each medicine. This course of treatment was chosen according to the guidance of WHO on measuring the affordability of medicines for chronic diseases [23]. The households' OOP is the amount of money that households have to pay after deducting the share of the health insurance organization (coverage). The price and insured co-payment were extracted from Health Insurance Organization reimbursement list 2019 to calculate patients' out-of-pocket (OOP) payments. It is important to note that almost all antidiabetic medicines have insurance coverage, and the reimbursement level is varied between 70%, 90% and 95%.

**Table 2:** The cost of medication therapy scenario and household OOP payments per treatment schedule

Medication therapy regimens		Treatment schedules	Treatment cost (30 days) *	Household OOP*	Share of OOP of treatment cost
**Mono therapy**		Metformin,	36,000	10,800	30%
		Glibenclamide	15,000	4,500	30%
		Gliclazide	24.000	7,200	30%
**Dual therapy**		Metformin + Acarbose	159,000	47,700	30%
		Metformin + Repaglinide	243,000	72,900	30%
		Metformin + Glibenclamide	123,000	36,900	30%
		Metformin + Gliclazide	132,000	39,600	30%
		Metformin + Pioglitazone	171,000	51,300	30%
**Triple therapy**	**A) Oral therapy**	Metformin + Pioglitazone + Glibenclamde	186,000	55,800	30%
		Metformin + Pioglitazone + Gliclazide	195,000	58,500	30%
		Metformin + Pioglitazone + Acarbose	222,000	66,600	30%
	**B) Oral therapy + Insulins**	Metformin + Insulin isophane	122,400	116,280	95%
		Metformin + Insulin glargine	306,900	291,555	95%
		Metformin + Insulin detemir	3,303,000	990,900	30%

^*^ Iranian currency (Rials)

The analysis was conducted using the budget share (formula 1) and capacity to pay (formula 2) methods for different rural and urban income deciles.

$$CPEs = \frac{monthly\;patien{t}^{^{\prime}}s\;OOP\;on\;anti-diabetic\;pharmaceutical\;expenditure}{monthly\;total\;houshol\; expenditure} >2\% \; formula\;1$$

$$CPEs = \frac{monthly\;patien{t}^{^{\prime}}s\;OOP\;on\;anti-diabetic\;pharmaceutical\;expenditure}{monthly\;Total\;houshol\; expenditure-food\;expenditure}> 8\%\;formula\;2$$

If the CPEs were more than 2% in budget share approach or more than 8% in capacity to pay approach, it was considered a catastrophic pharmaceutical expenditure.

## Results

The cost of treatment schedules, household out-of-pocket (OOP) while seeking those treatments, the catastrophic treatments, and the proportion of households to be confronted with CPEs in rural and urban areas for the year 2019 are presented in this section.

### The medication therapy cost and OOP payments in type 2 diabetes

Generally, clinicians choose three standard medication therapy regimens for managing type 2 diabetes. Table 2 shows the cost of treatment schedules and household OOP payments after deducting health insurance shares. However, it should be mentioned that these are the cost of initial treatments.

**Fig. 1:**
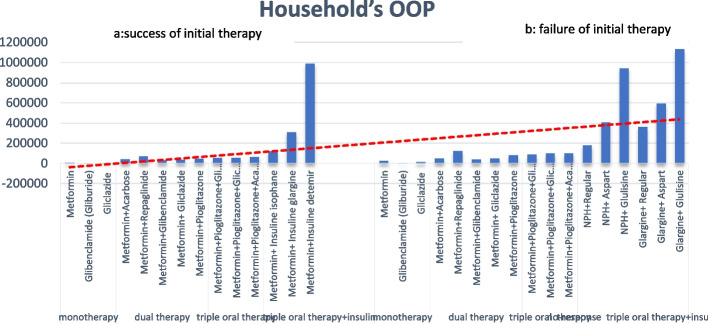
**a** shows the household's OOP payments in the case of success, and **b** shows the household's OOP payments in the case of failure of initial therapy

In case of initial treatment failure, clinicians may adjust treatment dosage or switch to the next medication therapy option. Figures 1a and 1b showed the household's OOP payments regarding the success and failure of initial therapy for each treatment schedule. The OOP payments increased significantly, particularly when Insulin was added to the schedule.

### The catastrophe antidiabetic medication therapy in different approaches

#### The budget share approach

**Fig. 2:**
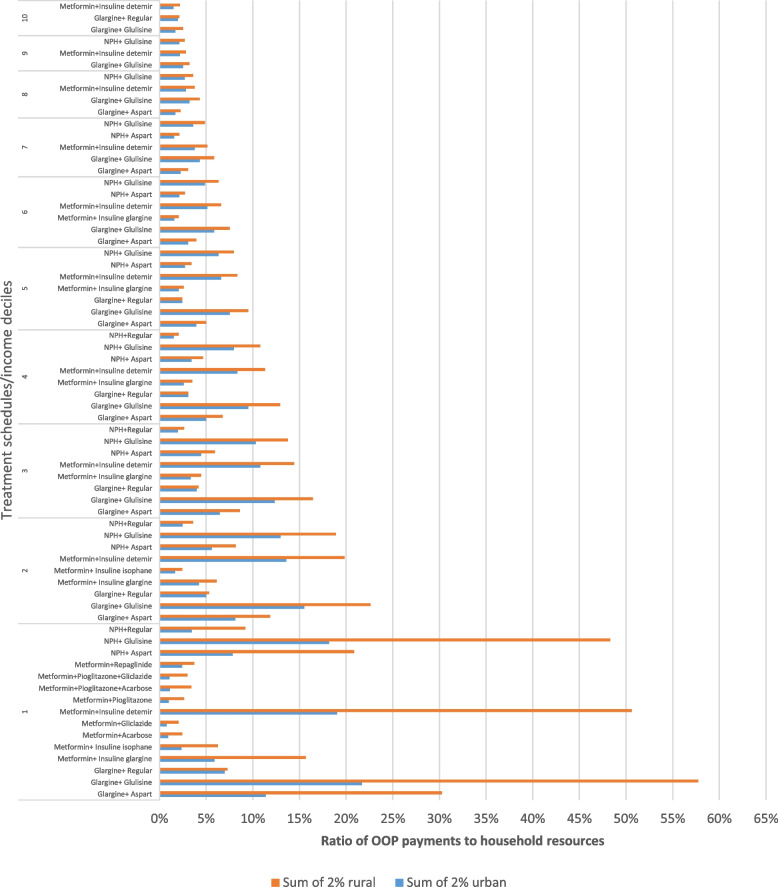
The catastrophe anti-diabetes medicines in each decile based on total expenditures

Figure 2 showed the catastrophic treatments when total household expenditures were considered a household resource. The antidiabetic medication costs were regarded as catastrophic if the ratio of OOP payments to household resources exceeded 2%.

It was observed that the catastrophe medication varied by treatment schedule and available household resources. The assessment showed that oral medication therapies were affordable, and the household incurred catastrophic expenditure when Insulin was recommended for disease management. Furthermore, there is a considerable difference between the shares of OOP payments between the households. The highest OOP payment share belongs to rural and urban households in deciles 1 to 3 for treatment schedules, including only insulins. By moving to wealthier households, some treatments were not catastrophic or catastrophic, with the share close to the benchmarked thresholds. Glulisine, detemir, and glargine insulin contributed significantly to increasing OOP payments due to high prices and lower insurance coverage compared with the other Insulin such as isophane insulin (with 70%, 70%, and 90% insurance coverage, respectively, compared to 95%). However, it is essential to note that exclusively insulin therapy (Basal + rapid or short-acting insulins) is prescribed for advanced type 2 diabetes patients when monotherapy, dual oral therapy, oral therapy and Insulin regimens in combination with oral medications fail. As a result, only a tiny percentage of patients with type 2 -almost 2%- were switched by clinicians to these treatment schedules.

The poorest households in deciles 1, 2, and 3 had the highest share of catastrophic medication therapy expenditures. Moreover, pharmaceutical expenditures for rural households were more catastrophic.

#### The household capacity to pay approach (Actual food spending)

**Fig. 3:**
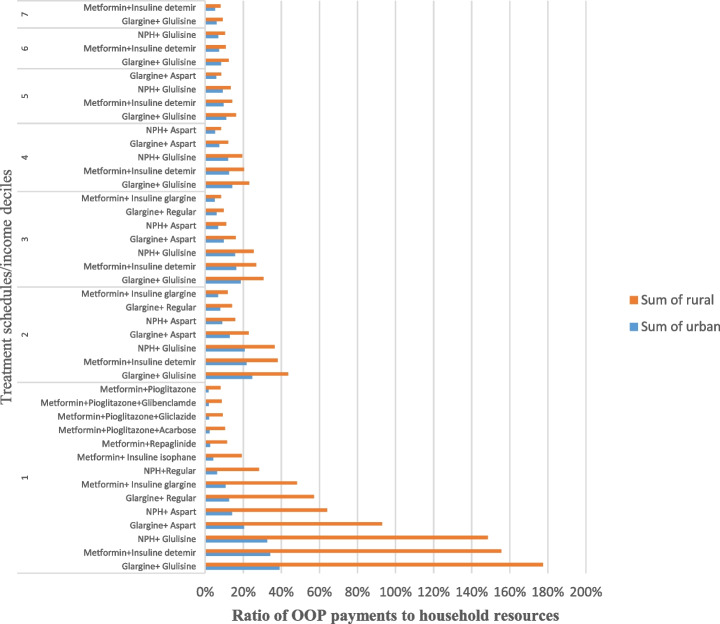
The catastrophe anti-diabetes medicines in each decile in Iran based on non-food expenditure

Figure 3 shows the treatments that put patients at risk of catastrophic pharmaceutical expenditures when household non-food expenditures were considered the household capacity to pay. The diabetes medication therapy was regarded as catastrophic if the ratio of OPP to household resources exceeded 8%. Our results show that all medication therapies were affordable for the wealthiest households in deciles 8, 9, and 10. The most catastrophe medication therapies belong to poor households in the first three deciles in rural and urban areas.

As mentioned later, catastrophic treatment schedules are exclusively insulin therapy prescribed to patients when there is not enough response to initial oral therapy with Insulin. The number of catastrophic therapies would further decrease by excluding this small group of patients. Generally, the number of catastrophic treatments was lower than the budget share approach (with a 2% threshold).

### The catastrophe antidiabetic medication therapy in rural/urban area

The proportion of households facing catastrophic payments for procuring antidiabetic medication is shown in Table 3. By treatment schedules, it varies from 0.0% in monotherapy to up to 50% in triple therapy with Insulin. Regarding residence type, the proportion of households in rural areas was higher compared to urban areas. For instance, in the 2% threshold, Metformin plus pioglitazone for 4% of rural households was catastrophic, while this proportion for urban households was zero. Additionally, the proportion of urban households using metformin plus insulin glargine is 24% lower. In the case of high price medicines with a lack of insurance coverage, such as insulin detemir and insulin glargine, the proportion of households confronting catastrophe pharmaceutical expenditure in rural (94%) and urban areas (90%) is high.

**Table 3:** The proportion of households facing catastrophic payments for procuring antidiabetic medicines in rural and urban areas

Scenario	Treatment schedule	Rural (2%)	Rural (8%)	Urban (2%)	Urban (8%)
**Monotherapy**	Metformin	0%	0%	0%	0%
	Glibenclamide	0%	0%	0%	0%
	Gliclazide	0%	0%	0%	0%
**Dual therapy**	Metformin + Acarbose	0%	0%	0%	0%
	Metformin + Repaglinide	4%	4%	0%	0%
	Metformin + Glibenclamide	0%	0%	0%	0%
	Metformin + Gliclazide	0%	0%	0%	0%
	Metformin + Pioglitazone	4%	0%	0%	0%
**Triple oral therapy**	Metformin + Pioglitazone + Glibenclamde	4%	4%	0%	0%
	Metformin + Pioglitazone + Gliclazide	4%	4%	0%	0%
	Metformin + Pioglitazone + Acarbose	4%	4%	0%	0%
**Oral therapy + Insulin**	Metformin + Insulin isophane	4%	4%	4%	0%
	Metformin + Insulin glargine	46%	12%	22%	4%
	Metformin + Insulin detemir	94%	56%	90%	31%

Finally, in the "capacity to pay approach," the proportion of households with catastrophic expenditure is lesser than the budget share. This measurement approach difference should come to attention while making the policies because they may be over or under-estimated.

## Discussion

In this study, the catastrophic pharmaceutical expenditures of diabetes medicines are being comprehensively investigated in different treatment schedules. The budget share and capacity to pay approaches were used to explore the catastrophic treatments and the incidence of catastrophic financial effects among rural and urban Iranian households. The new thresholds for the pharmaceutical sector were set considering the share of pharmaceutical expenditures (PE) to total health expenditures in Iran.

To the best of our knowledge, the present study can be mentioned as the first to assess medication therapy's catastrophic expenditures in different mono-and combination treatment schedules. In the previous studies, only a limited number of medicines were assessed [8, 13, 15, 17, 24, 25], while this study covers a range of treatment schedules.

The results showed that the OOP payment increased with increasing doses of medicines in initial treatment or when switched to second-line options. However, the treatment is still affordable. No one has encountered catastrophe expenditures with mono-and dual and sometimes triple oral therapies, even in rural areas. These findings align with Amiresmaili and Emrani's work which showed that only a tiny fraction, 0.007% of Iranian families, were required to pay over 40% of their income for Metformin [15]. In addition, Zaheer et al. study revealed that generic essential diabetes medicines in 17 surveyed countries were affordable except in a few countries in the low-income group, including Tanzania [26].

Treatment schedules were catastrophic when Insulin was added to oral medication therapies. In line with the previous studies, Insulin is a significant contributor to catastrophic expenditures [27–29]. According to the Prospective Urban Rural Epidemiology (PURE) study at 604 communities in 17 low-, middle-and high-income countries around the world, Insulin was the least affordable medicine [27]. This study used micro-level data. The consistency of our results with such studies encourages promoting a macro-level assessment. This would help to compare different time scales and assess different diseases.

The lowest-income households in rural areas are at most risk of being pushed toward a catastrophic border [27]. This shows where to target and aim to improve access for more vulnerable households, mainly where budget impacts are low. It is also essential to consider other factors such as treatment pathways, consumption and volume of medicines, and potential budget impact.

Different studies confirmed that rural residences were significantly at risk of CHEs [30–33]. This may be due to their low income, low education level, or delay in disease diagnosis [34, 35]. According to the results of the latest population and housing census (2016), the rural population is less than the urban population in Iran. In better words, 74% of the country's population lives in urban areas and just 26% in rural areas [22]. Meanwhile, a wide socio-economic gap caused by extreme urbanization has also been reported in urban areas of the country [36].

The results showed that more catastrophic treatments were observed in the budget share approach, and a higher proportion of households faced catastrophic expenditures. Cylus et al. [18] showed the difference between standard methods and emphasized the overestimation effects of the "budget share method." This is in line with the results presented in the current study.

Furthermore, Niëns et al. [21] showed that variations in the thresholds of catastrophic payments could lead to significant discrepancies in the results. If individuals were permitted to consider no higher than 1.0% of their daily income on glibenclamide, it would not be affordable to over 99% of the people; however, if the threshold is increased to 10%, glibenclamide would not be affordable for over17% of the people [21]. There are concerns because deliberate changes in threshold levels would seriously influence estimations on affordability along with its impact on the policymakers [12]. Defining adjusted thresholds for different parts of health expenditure may be the best option for developing policies to protect more individuals. Niëns et al. (Year) encouraged attempting to deal with an established mixture of methods and thresholds regarding affordability [8].

Mszar et al. revealed that although having diabetes increases the odds of financial hardship, individuals with concurrent atherosclerotic cardiovascular disease and diabetes had the highest relative odds of expressing an inability to pay at all when compared with those with neither of the condition (odds ratio, 2.69; 95% CI, 2.21–3.28) [37].

### Limitations

In using macro-data, the mean expenditure in each income decile was used while assuming the linearity of income. While the distribution of income in each income group could be skewed since most individuals in the group could be potentially in the lower income bracket than the average. The average income for each income may be overestimated, and thus, the catastrophic effects of the medicines may be underestimated in the analysis. In addition, although using macro-level data is simple and generalizable, there is no access to individual-level data, so it is not possible to provide further analysis based on the actual status of each household like population income status, household size etc.

## Conclusions

Poorer households and patients who are resistant to first-line therapies probably suffer more from catastrophic expenditures as they will not be able to afford the Second- and third-line antidiabetic treatment expenses. Though this could be a small group of patients in Iran, making more efficient reimbursement decisions is required to ensure the affordability of antidiabetic medications.

At a policy level, to provide policymakers with a better understanding and more profound insights toward catastrophe expenditure within health systems and across countries, defining threshold value for assessing catastrophic expenditure for each component of health expenditure and using the conventional approach of aggregated data is strongly recommended.

## Data Availability

All data is available and can be provided by the corresponding author upon reasonable request.

## Abbreviations

OOP: Out-of-pocket
UHC: Universal health coverage
CHEs: Catastrophic health expenditures
CPEs: Catastrophic pharmaceutical expenditures
LMICs: Low-and middle-income countries
NCDs: Non-communicable diseases
HTP: Health transformation plan
WB: World bank
WHO: World health organization
SDGs: Sustainable development goals
SCI: Statistical center of Iran
